# Sustainability of Rearing System Using Multicriteria Analysis: Application in Commercial Poultry Production

**DOI:** 10.3390/ani11123483

**Published:** 2021-12-07

**Authors:** Lucia Rocchi, Alice Cartoni Mancinelli, Luisa Paolotti, Simona Mattioli, Antonio Boggia, Francesco Papi, Cesare Castellini

**Affiliations:** 1 Department of Agricultural, Environmental and Food Science, University of Perugia, Borgo XX Giugno 74, 06124 Perugia, Italy; lucia.rocchi@unipg.it (L.R.); luisa.paolotti@gmail.com (L.P.); simona.mattioli@unipg.it (S.M.); antonio.boggia@unipg.it (A.B.); cesare.castellini@unipg.it (C.C.); 2 CARNJ Società Cooperativa Agricola, Via Martiri della Libertà, 27–60035 Jesi, Italy; f.papi@fileni.it

**Keywords:** one welfare, multicriteria analysis, poultry production

## Abstract

**Simple Summary:**

Organic poultry production is growing annually in Europe and USA. The main objective of the organic rearing system is to improve animal welfare, environmental impact and human welfare. All of these aspects are part of the "One Welfare" approach, which also includes food security, sustainability, the reduction of human suffering and improvements in the productivity of farms by applying high welfare standards. However, “One Welfare” is still a theoretical concept; it is important to determine practical applications for this concept in order to evaluate the production system in its entirety. This study, for the first time, applies the One Welfare approach in commercial poultry production by developing a specific a multicriteria model. This model was used to compare three different rearing systems, considering all their inputs and outputs simultaneously.

**Abstract:**

The aim of the present study was to develop a multicriteria model for the comparison of three commercial poultry farms: organic with Ross 308 genotype (OR), organic with Naked Neck genotype (ONN) and a conventional system (C), which represents the most common commercial farming system. A model based on multicriteria decision analysis was developed, considering for the first time the One Welfare approach in an operational manner, including three dimensions: human, environmental and animal welfare. The three alternatives demonstrated different performances, according to the different dimensions considered. In particular, the two organic systems performed better for human welfare and animal welfare, with relevant differences due to the genetic strains used. Conventional rearing performed better for the environment index due to the method chosen. The multicriteria analysis showed that the organic system performed better overall than the conventional system. In particular, the use of an adapted Slow Growing (SG) strain positively affected the final rank, mainly by reducing welfare problems and producing good economic and social performance. The stability of the results was verified by performing a sensitivity analysis, specifically a weight stability analysis, which confirmed the strength of results.

## 1. Introduction

According to the Eurostat 2019 report, the EU’s organic production over the period 2007–2017 increased by 5.6% per year. The main countries involved were Spain, Italy, France and Germany. Despite the rapid growth of the sector, organic animal production represents only 3% of the total. This is probably due both to the high cost of feed and animal medication restrictions.

Nevertheless, organic poultry production demonstrated a higher annual growth rate than other forms of livestock production. The Organic Rearing System (ORS) consists of farm practices that contribute to the preservation of natural resources and is also based on higher animal welfare standards (IFOAM principles).

**Figure 1:**
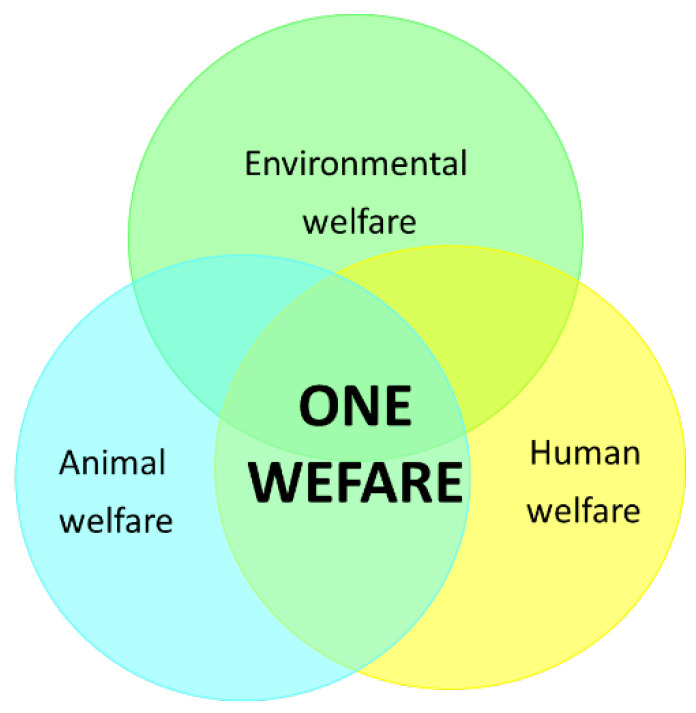
One welfare concept.

Therefore, the main pillars of organic production animal welfare, environmental impact and human welfare are contained within the current concept of “One Welfare”, which is becoming very innovative in productive chains (Figure 1). In fact, the One Welfare concept is based on the promotion of direct and indirect links between animal welfare with human welfare [1] by considering environmentally friendly farm systems. Moreover, One Welfare also supports other important global issues, such as: food security, sustainability, reducing human suffering and improving productivity within the farming sector by applying high welfare standards.

Despite the strict rules concerning ORSs in the EU and in USA, these systems are still very heterogeneous. This is probably due to the fact that such regulations are mainly focused only on some aspects, such as forbidding the use of synthetic products in animals feed, prohibiting the use of antibiotics and applying a minimum animal slaughter age.

In the organic poultry sector in particular, some aspects are only recommended, such as the use of local feed ingredients, the use of suitable poultry strains and the presence of pasture in outdoor runs.

One of the main characteristics of ORS is the use of outdoor space by animals and, consequently, pasture availability. Many studies [2,3,4,5] reported that only some poultry genotypes are suitable to be reared in free-range conditions; moreover, the grazing capacity of animals positively affects the quality of their products [6].

The current EU regulation allows the use of different chicken genotypes but does not make mandatory the use of slow-growing (SG) chicken breeds, which are the most appropriate for ORS. By contrast, it is well known that fast-growing (FG) chicken genotypes, due to their rapid growth and high breast yield, are not suitable to be reared in ORS [7]. Indeed, the use of an appropriate poultry strain is crucial to maintaining the good welfare and health status of birds along the full period of rearing (81 d) and it is essential for assuring the use of outdoor runs.

Thus, the choice of suitable poultry genotypes for ORS is still an open issue and the use of different genotypes increases diversity among the ORSs in the world.

Indeed, the ORS represents a complex production system because it is constituted by many factors, which are still not standardized. For this reason, in order to compare different rearing systems, all the inputs and outputs involved should be evaluated not as single traits but as a whole, through a multifactorial approach.

Previous research features a number of studies comparing the effect of ORS on single aspects (economy and productive performance, animal welfare, environmental impact and qualitative characteristics); only a few of them analyzed all the results together [8], and these were mainly restricted to experimental farms.

The aim of the present paper is to develop a multicriteria model for the comparison of sustainability of conventional vs. organic commercial poultry farms. In particular, the analysis considered two different organic systems, differing for the genetic strains used (organic with Ross 308 genotype-OR, which is a fast-growing type and organic with Naked Neck genotype-ONN, which is a slow-growing type), plus a conventional system (C), representing the most common rearing system across commercial farming. Moreover, this study attempted for the first time to develop the One Welfare concept in an operational way for broiler rearing systems.

## 2. Materials and Methods

### 2.1. Description of the Systems Analyzed

Three types of poultry systems were examined: a conventional intensive system and two organic systems, featuring different genetic strains (Ross 308 and Naked Neck genotype, respectively). The farms analyzed were commercial poultry farms (*n* = 2 similar per type; total 6) located in the same area of central Italy. Table 1 presents the main structural and management characteristics of the farms (mean data).

The conventional system featured a standard broiler rearing system, using meat-type birds, concentrated feed and controlled housing (artificial light and climate control, automatic water and feed supply). The birds were raised in this system according to the Directive 2007/43/EC for the protection of chickens kept for meat production.

In Europe, Commission Regulation No 848/2018 regulates organic systems for poultry and livestock production. Following this regulation, the organic systems in our study used meat-type birds, organic feed and controlled housing. Moreover, in the organic system it is mandatory that poultry have access to an open space for at least one third of their life. Therefore, the facilities in organic systems must provide an outdoor area with the presence of pasture. Table 2 reports the main ingredients used in the concentrated feeds.

### 2.2. Criteria Considered in the Analysis

In order to compare the sustainability of the three systems within in the framework of the One Welfare approach [1] by means of a multicriteria analysis, a set of different criteria was identified. As One Welfare is a complex concept, the evaluation framework was organized in a hierarchical way, in order to maintain such a complexity in the analysis (Table 3). Initially, three dimensions were considered: animal welfare, human welfare and environment [1]. Next, for each dimension all the relevant categories describing it were defined. The criteria derived from the categories; sometimes, the categories and criteria coincided (for instance, environmental categories and criteria). The criteria were chosen according to previous studies [8,9,10,11,12], aiming to fulfill all the relevant categories in each dimension of the One Welfare concept. Section 2.2.1 to 2.2.3 describe in detail all the criteria in each dimension.

#### 2.2.1. Animal Welfare

The animal welfare traits were assessed through the evaluation of animal behavior with the use of a computerized system (Noldus Technology, Wageningen, The Netherlands); moreover, an evaluation of the podal and sternal lesions on 50 animal carcasses/farm/system was performed at the slaughterhouse.

The behavioral aspect was assessed by positioning in advance one camera inside the shelter on each farm; for the organic systems, one camera was also located in the outdoor space, at a 5 m distance from the shelter. The behavior was investigated one week before the slaughter (37 d and 74 d in the conventional and in the organic system, respectively). Three 20 min videos were recorded for each group of farms through the activation of the cameras remotely. The videos were analyzed by an expert observer through a pre-defined ethogram (Table 4), using the instantaneous scanning sampling method [13].

The behavior frequency was observed for each animal and then the percentage of time dedicated to each specific behavior was calculated [4].

The behaviors recorded were divided into three main categories: activity (walking, rest, roost), eating (feed, grass, drink) and comfort. Concerning the use of the external pasture, only the walking activity and the feeding behavior (grass) were selected for the multicriteria.

Secondly, the presence or absence of sternal and footpad lesions were evaluated at slaughtering (*n* = 50 carcasses/farm). For each category, the percentage of animals was calculated by modifying the method reported by Berg [14].

The final indicators (criteria) used for monitoring animal welfare were: use of external pasture (only for the ORS), kinetic activity, feeding, comfort and sternal and podal lesions.

#### 2.2.2. Human Welfare

The human welfare category includes aspects relevant for a fair satisfaction of farmers, citizens and consumers. Therefore, the criteria included are linked to the social and economic dimension of the sustainability framework. The social criteria are related to the work conditions and consumers’ expectations of nutritive meat characteristics, while the economic criteria include performance and classic economic indicators. More specifically, the criteria covered five categories: meat nutritive quality, antibiotic resistance, work condition, economic and productive performance. Details on each topic are provided below.

Meat quality: after slaughtering, the breast muscle (15 breasts/per farm/system) was separated from carcasses and transported to the laboratory for chemical analysis.

The fatty acid profiles were extracted from the meat, following the method described by Folch et al. [15]. The fatty acid composition was determined using a Varian gas chromatograph (CP-3800), equipped with a flame ionisation detector and a capillary column of 100 m length × 0.25 mm × 0.2 μm film (Supelco, Bellefonte, PA, USA). Helium was used as the carrier gas, with a flow of 2 mL/min. The split ratio was 1:80. The oven temperature was programmed at 40 °C and held for 1 min, then increased up to 163 °C at a rate of 2 °C/min, held for 10 min, increased up to 180 °C at a rate of 1.5 °C/min, held for 7 min, increased up to 187 °C at a rate of 2 °C/min held for 2 min, and finally increased up to 230 °C at a rate of 3 °C/min, held for 25 min. The injector and detector temperatures were set at 270 °C and 300 °C, respectively. Individual FAMEs were identified by comparing the relative retention times of the peaks in the sample with those of the standard mixture (FAME Mix Supelco). The fatty acids were expressed as % of total fatty acids. The average amount of each fatty acid was used to calculate the sum of the total polyunsaturated (PUFA) acids from the n-3 and n-6 series.

Antibiotic resistance: the antibiotic resistance of the gut flora in slaughtered poultry has been inferred from a scientific study performed on the same farms [16]. The percentage of multi-resistant isolates in the different production types was used.

Work safety: the level of safety at work was investigated, analyzing the presence of collective and individual protective devices and counting the registered work accidents. Moreover, the chemical risk (e.g., respiratory or dermatological diseases due to the use of chemicals), biological risk (e.g., in case of high microbial loads or potential vectors of zoonosis) and physical/mechanical ones to which workers are subjected were considered, accounting the hours of work required for the performance of the typical duties. In fact, the hours of work necessary to carry out the daily activities are proportional to the probability of the aforementioned risks occurring. The two criteria in the work safety category (risk category and work load) were estimated on the farm through a check-list questionnaire for a qualitative evaluation.

Economic performance: this category provides general information on direct costs as well as on farm revenues. The calculation was based on the data provided by the company.

Production performance: the criteria in this category can be considered as indirect economic information. Specifically, the performance indicators considered were: the final weight achieved at slaughtering, the food conversion index (FCI) (calculated by the kg feed/kg meat produced ratio) and the mortality rate considered at the end of the cycle (expressed as dead birds/number of initial birds). All the data were directly collected on farm.

#### 2.2.3. Environment

The environmental impacts were evaluated by means of a life cycle assessment (LCA), examining 5 of the 11 categories using the Ecoindicator method [17], specifically: respiratory inorganics, climate change, acidification/eutrophization, land use and fossil fuels.

The classic four phases, goal and scope definition, life cycle inventory, life cycle assessment and interpretation, established by the standards ISO 14040 and ISO 14044 [18,19], were performed. The system boundaries ran from the production of the initial necessary inputs (cultivation of the main feed ingredients) to the rearing phase (production of poultry), including the intermediate feed manufacturing and transport processes (LCA from cradle to gate). The functional unit considered in all the systems was 1 kg of poultry meat.

Concerning the foreground data, the main operational techniques for producing the cultivations destined to the feed ingredients, together with information about provenience of raw materials and covered transport distances, were directly collected from the farms involved in the case studies. Furthermore, the amount and composition of the different feeds were collected in the same way. The farms provided data on emissions in air related to the transformation process from cultivation to feed, and subsequently all the other main data related to the poultry rearing (e.g., water, electricity, gas consumption, number of birds reared and final amount of meat produced) for each case study.

The other general data (background data about transport, fuel consumption, generic materials production and electricity sources) involved in all the phases of the life cycles, were taken from the Ecoinvent database 2.2 [20].

The data related to the main emissions along the life cycle can be divided into emissions occurring during the cultivation phase, emissions during the transformation from cultivation to feed and emissions occurring during the rearing phase. In relation to emissions coming from the cultivation phase, nitrous oxide emissions from fertilizers were calculated using Global Nitrous Oxide Calculator [21], an online tool that returns the output data after entering information on the environment, agronomic management and geographical location [22]. Ammonia emissions were considered equal to 15% of the total nitrogen used [23]; CO_2_ emissions were considered equal to 20% of the total urea used [24]. The emissions for transforming cultivation in feed mainly consisted in particulates and organic substances and, as mentioned above, they were directly collected from the farms of the case studies. We used the IPCC BREF average values to determine the rearing emissions [25], as well as the NH_3_ emissions from manure and bird housing.

#### 2.2.4. Data Processing

The mean values of animal welfare, chemical characteristics of meat and LCA values were analyzed with a linear model considering the effect of the three rearing systems (C, OR and ONN), using the Stata package [26]. These values were used for the successive multicriteria analysis.

#### 2.2.5. Multicriteria Analysis

**Figure 2:**
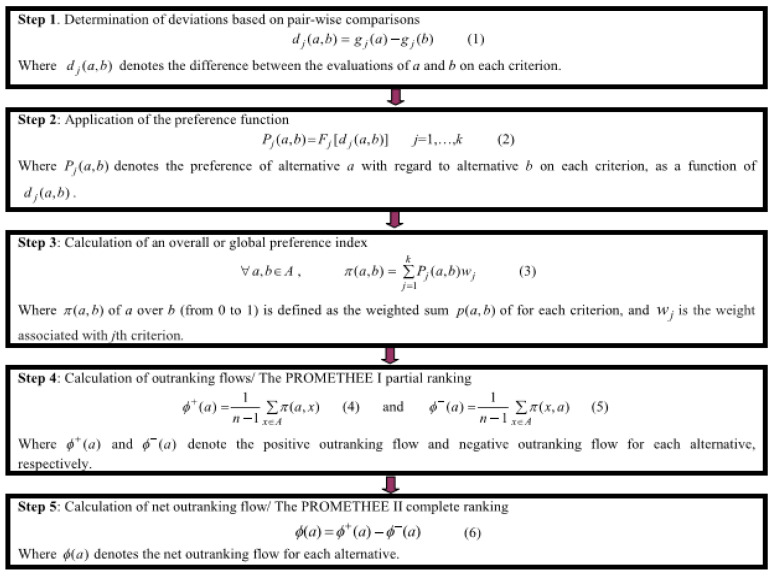
Stepwise procedure for PROMETHEE I and II (source: [31]). *a*, *b*: alternatives; *j* = 1, …, *k*: criteria; *gj*(*a*) and *gj*(*b*): values; *P*: preference relation; *Fj*: preference function; *w_j_*: weight associated to the *j*^th^ criterion; *π* (*a*,*b*): global (weighted) preference of criterion *a* over criterion *b*.

To rank the three rearing systems (two organic, one conventional), a multicriteria analysis was performed, using the criteria described in Section 2.2 and applying the PROMETHEE I (partial ranking) and PROMETHEE II (complete ranking). PROMETHEE [27,28,29] is a family of outranking multicriteria methods, applied to very different research fields, including the sustainability analysis of rearing systems [8]. PROMETHEE is a method based on the pairwise comparison of alternatives, with weights representing the coefficient of importance; the method is not totally compensatory; therefore, poor results on an indicator cannot be counterbalanced by good results on another [30]. The steps to perform the method are presented in Figure 2.

As demonstrated in Figure 2, the outputs of the PROMETHEE I are two outranking flows: the positive outranking flow (Phi + (a)) represents the outranking power of an alternative over all the others: the higher the Phi + (a), the better the alternative [29]. The negative outranking flow (Phi − (a)) measures the weakness of an alternative, and how much it is outranked by all the others: the smaller the Phi − (a), the better the alternative [29].

PROMETHEE II produces the Net outranking flow Phi(a), which is a measure of the balance between positive and negative flows. The higher the net flow, the better the alternative. In real-world applications, it is recommended to apply both PROMETHEE I and II, as the complete ranking is easier to read and use; however, the partial ranking can be useful to finalize a proper decision [29]. In this paper, both PROMETHEE I and II were applied, first to all the criteria for a whole valuation, and then to each dimension (Animal Welfare, Human Welfare, Environment). The weights were considered equal across the criteria.

#### 2.2.6. Sensitivity Analysis

All the results obtained were submitted to a sensitivity analysis, with the aim of testing the stability of the results, with particular regard to the variation of weights, since in this application they were the same for all the criteria.

We performed a weight stability analysis by testing the sensitivity of the ranking to change when a different weight would be attributed to different criteria [32], looking for reversal points (i.e., changes in the relative positions of two cases in a ranking). This methodology has been widely studied in numerous multicriteria decision aid methodologies [33] and it recurs in pairwise MCDA methods, such as PROMETHEE [34].

The weight stability analysis was performed using the visual stability interval, which highlights how the final ranking varies as a function of the weights of a single criterion [35]. Only the global ranking was considered. The stability intervals help to understand how much (in percentage) a weight needs to change in order to reverse the final rank.

## 3. Results

Table 5 reports the set of criteria that was developed, representing the performance of the three poultry systems to be used for MCDA. The first column reports the name of the criteria, followed by the unit of measurement and the specification of whether the goal was to maximize or minimize each criterion. Next, the values for the three rearing systems are reported.

### 3.1. Animal Welfare Performance

For the animal welfare criteria, the C system exhibited the highest percentage of animals in feed behavior compared to OR and ONN. Although it featured the same rearing system, the ONN exhibited the highest percentage of animals performing high kinetic activity and using the outdoor space, with respect to OR (21.8% and 43.7% vs. 10.9% and 20.2%). The podal and sternal lesions were higher in the OR and C systems.

### 3.2. Human Welfare Performance

The productive performance parameters considered in the human welfare criteria displayed the best live weight in the OR system, whereas the C system was more efficient in terms of mortality rate and feed conversion index. The economic parameters demonstrated that the costs were higher in the organic rearing systems in comparison to the conventional. By contrast, the revenue was higher in the organic systems. In particular, OR exhibited the best performance according to both traits.

Among the three systems, no particular differences related to the meat quality characteristics (n-6 and n-3 PUFA) were found. The indicators risk category, work load and antibiotic resistance exhibited a similar evolution: the two organic systems performed the same, while the conventional system diverged significantly. Among the three, only work load was better in the C system.

### 3.3. Environment Performance

In reference to the environmental indicators, the conventional system demonstrated the greatest impact in respiratory inorganics, climate change and acidification/eutrophication, while it produced the best score for the land use category. The ONN system was the system with the lowest impact in relation to respiratory inorganics and acidification/eutrophication, while it demonstrated the highest impact for land use and fossil fuels. At the same time, the OR system performed best for climate change and fossil fuels. Therefore, there was no homogeneous result in terms of the best performances for all the categories.

### 3.4. Multicriteria Analysis

**Figure 3:**
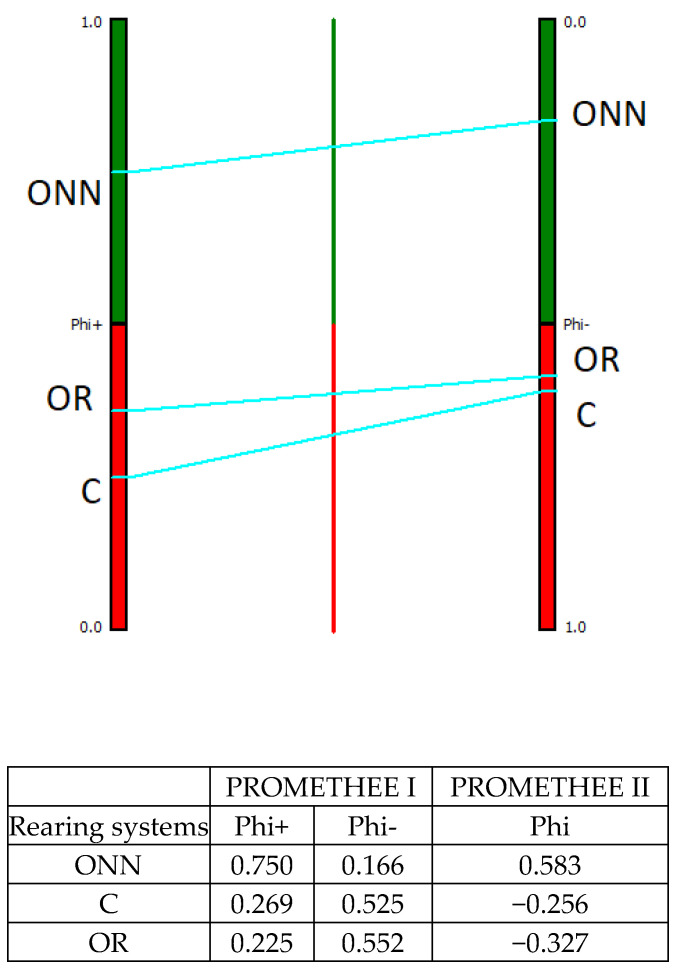
Animal welfare results according to PROMETHEE I and PROMETHEE II. ONN organic Naked Neck; OR organic Ross; C, conventional.

**Figure 4:**
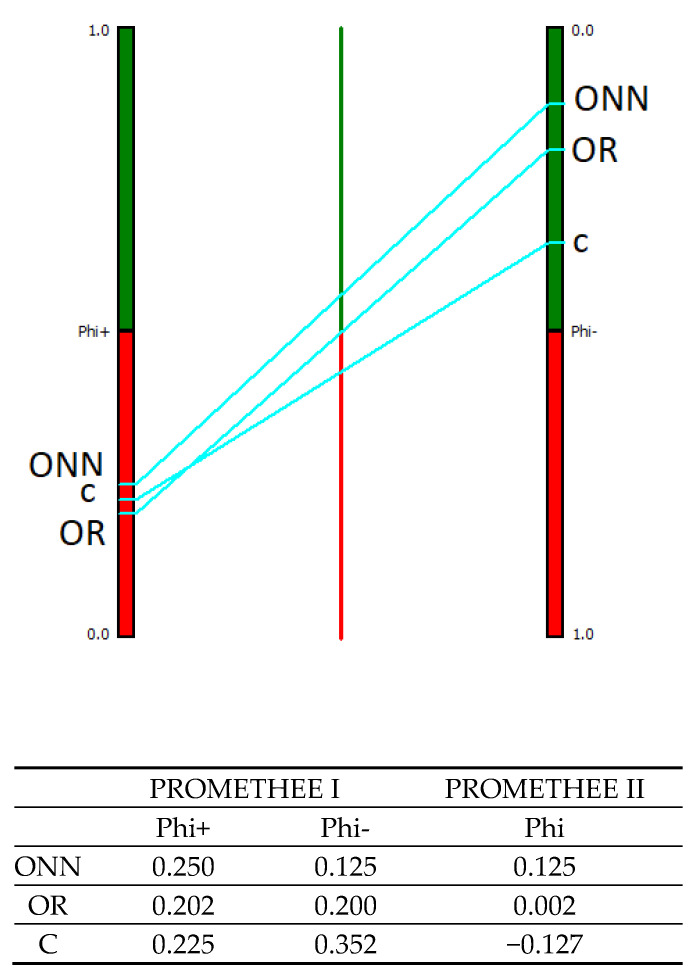
Human welfare results according to PROMETHEE I and PROMETHEE II. ONN organic Naked Neck; OR organic Ross; C, conventional.

**Figure 5:**
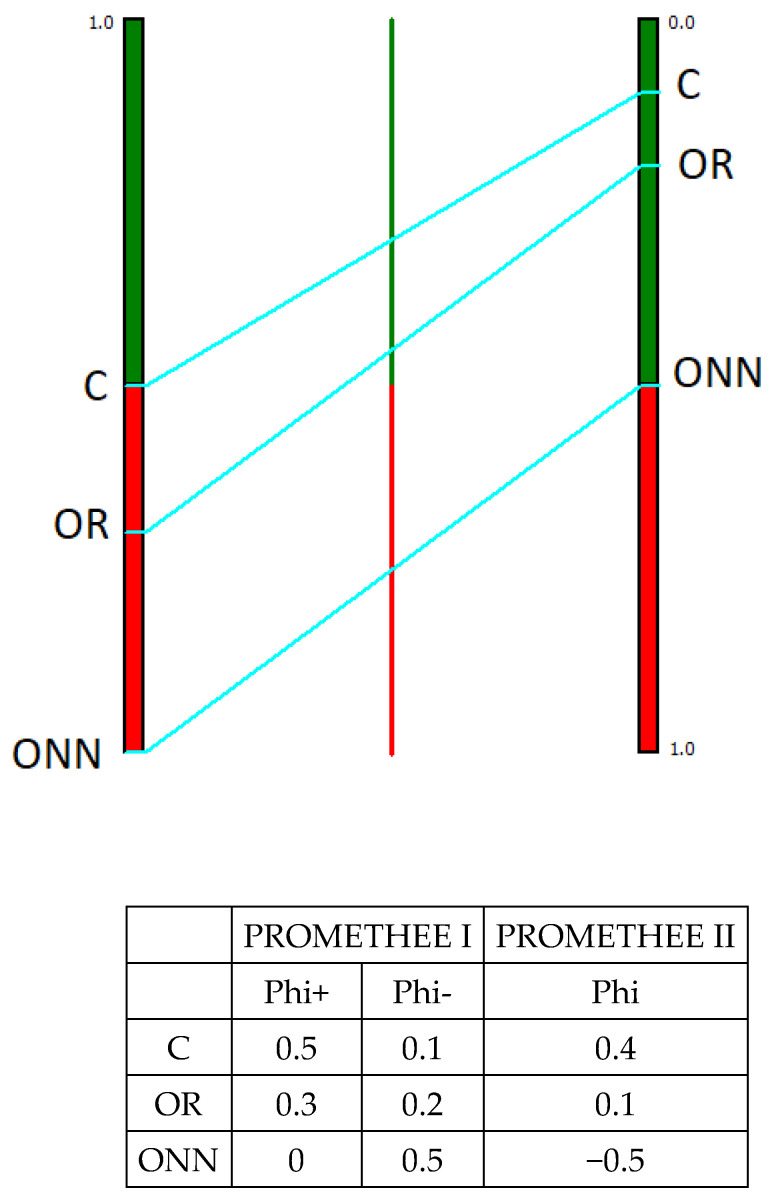
Environment results according to PROMETHEE I and PROMETHEE II. ONN organic Naked Neck; OR organic Ross; C, conventional.

**Figure 6:**
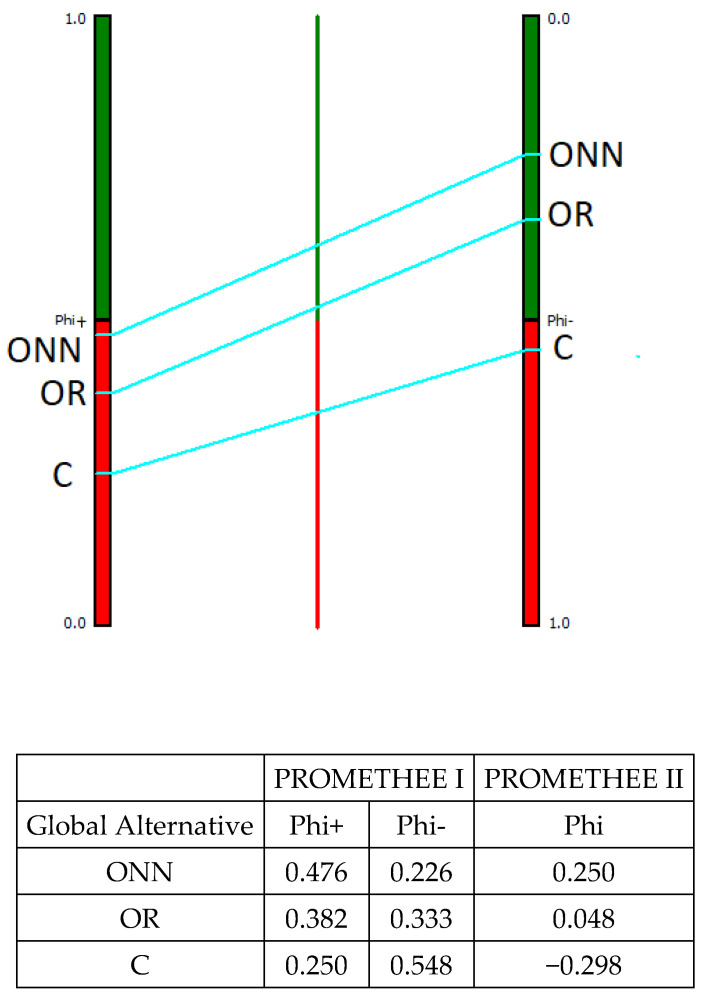
Overall results according to PROMETHEE I and PROMETHEE II. ONN, organic Naked Neck; OR, organic Ross; C, conventional.

The MCDA analysis using the PROMETHEE method provided two different rankings for each alternative: a partial ranking using PROMETHEE I and a complete ranking using PROMETHEE II. Figure 3, Figure 4, Figure 5 and Figure 6 display the results: the figures provide only the PROMETHEE II output (Phi; complete ranking), while the results of the PROMETHEE I (Phi+ and Phi-; partial ranking) are in the table below each figure, along with the numeric results of the PROMETHEE II. The figure of the PROMETHEE II corresponds to the net value, which is equal to the difference between the two flows (Phi): a position on the green part of the bar means a good ranking, while a position on the red part means a poor ranking; moreover, the higher the position of a system, the better its ranking. Looking at the numeric output in each table, the results of the PROMETHEE I always a feature positive sign: however, a high value for the Phi+ means a good result for an alternative, while a high value for the Phi- means poor performance. The complete ranking (Phi, indicated in the figures) features a sign: therefore, a positive sign is for good results and negative is for poor ones.

In reference to the animal welfare category (Figure 3), the multicriteria analysis results demonstrated the clear superiority of ONN in comparison to the other two alternatives, which presented negative net values. Even considering the Phi^+^ and Phi^-^ values, the ONN resulted in much higher Phi^+^ and much lower Phi^-^ in comparison to OR and C. These two rearing systems exhibited similar values in terms of Phi^-^, and relatively close values in terms of Phi^+^, probably due to the use of the same genetic strain.

The human welfare results are presented in Figure 4. The three alternatives are quite close in such a dimension, in terms of both the complete ranking (Phi) and the positive flow (Phi^+^) values. However, the best alternative was the ONN, followed by OR and C, based on the complete ranking. Moreover, the conventional system was the only alternative with a negative net value. In terms of Phi^+^, the best alternative was again ONN, followed by C and OR, while in terms of Phi^-,^ the conventional system performed much worse than the other two, and this is probably the reason why it was the lowest in the complete ranking.

Finally, in relation to environment (Figure 5), unlike in the previous rankings, the conventional system was the best alternative, followed by OR and ONN. This was related to the good feed conversion efficiency of this system, which reduced some environmental impacts, such as land use. The difference with ONN, which displayed a negative net value, is clear, while OR presented an intermediate position, probably because it used the same fast-growing strain as the conventional system. In ONN, the positive flow Phi^+^ was 0, while the negative flow Phi^−^ was clearly higher than in the other two alternatives.

In relation to the overall evaluation obtained (Figure 6), the ONN system was found to the best alternative, in terms of both its partial and complete ranking, followed by OR and then by C. In particular, ONN demonstrated both the best positive and the best negative outranking flows; this resulted in a positive Net flow. The OR demonstrated a Net outranking flow value very close to 0, because it performed worse in both the negative and the positive flow compared to ONN. The conventional system performed poorly in both the indexes Phi^−^ and Phi^+^ and was therefore the worst alternative based on the Phi (complete ranking).

### 3.5. Sensitivity Analysis Results

The results of the sensitivity analysis are presented in Table 6 for the weights that featured rank reversal; all the other weights did not feature rank reversal, meaning that the rank remained the same whatever the value of those weights.

In particular, the first column of the table reports the stability interval between the two organic systems (switching between the first and second positions), the second column reports the stability interval between OR and C (switching between the second and third positions), while the third column reports the interval between OR and C, (switching between the first and third positions).

Animal welfare was the dimension with the fewest reversal points. In particular, ONN remained the best alternative in comparison to the others, except for comfort and podal lesions, which caused reversal points with OR and C, respectively. Podal lesions also caused a reversal point between OR and C, along with sternal lesions.

The stability of these results can be associated first of all with the different genotypes used (ONN vs. OR) and then with the typology of rearing (organic vs. conventional).

By contrast, human welfare presented the largest number of reversal points. In particular, in the two organic options, there were just two “no rank reverse” out of ten criteria, while the reversal points versus the C were four with OR and five with ONN. However, there were several criteria with a reversal point only in the case of a very high increase in the singular weight (100%), with all other aspects remaining unchanged. In this case, we can say that the most important factor in stability was the rearing system, instead of the genotype, although the different strain rewarded ONN more in the final ranking.

The environmental dimension was the one in which the C system performed best. However, C featured rank reverse with ONN in only two cases, land use and fossil fuels, which are the two criteria strictly linked to the feed conversion efficiency: a low feed conversion efficiency in broilers implies that more land and fossil fuel are necessary to produce feed ingredients. It is reasonable to believe that in this case, the most important factor in the reversal points was the genetic strain. Between OR and C, which shared the same genetic strain, there was just one rank reverse (fossil fuel), which was linked to the diet and therefore to the different rearing systems. Moreover, ONN exhibited three reversal points with OR, adding climate change to the two already mentioned.

## 4. Discussion

The three rearing systems exhibited different performances, according to the dimensions considered. As previously affirmed, multicriteria analysis permits the comparison of performances according to single criteria and to place these performances into some sort of picture. This effort is crucial for developing a robust framework for the One Welfare concept. Only a few papers used multicriteria analyses for comparing different poultry rearing systems, and to our knowledge this is the first paper to use data from commercial poultry farms.

Our multicriteria analysis demonstrated that the conventional rearing system generally featured poor values for the animal and human welfare indexes, while it performed best in the environment index. These results can be explained by the method used for environmental valuation; in fact, the LCA approach is designed for emphasizing the productive performance of the systems; thus, techniques using high productive strains (C and OR) display better LCA performance [36,37,38,39,40]. In any case, the longer rearing period of organic (81 vs. 42 d) produced a greater environmental impact, while the higher animal density of the conventional system does not couple with significantly higher impact [41].

As previously pointed out [12,42], some differences in environmental impact between organic and conventional systems are still rather difficult to be integrated into LCA. Therefore, the results of the LCA practically lack information about the effects on biodiversity and soil quality or the multifunctionality of agriculture [38]. The environmental impact of organic systems requires an analysis beyond the LCA approach, including other crucial aspects. Indeed, detailed analysis assessed with different methods, such as the emergy and comparing experimental case-studies suggest the better performance of organic and free-range systems [8,9,40,43,44,45,46]. In any case, independently of how environmental impact is measured, the feed used (the type and origin of ingredients and their levels of renewable energy) is one of the main sources of impact [41].

The two organic systems demonstrated better performances in animal welfare, with relevant differences due to the different genetic strains used (Ross 308 vs. Naked Neck). The SG strain (ONN) featured a better animal welfare Index. The organic farms using the SG strain overwhelmed the other two groups (conventional and OR) using the FG strain: in this case, the rearing system exerted a minor effect.

Many studies reported that the FG strain, due to its fast growth, is not suitable for organic systems. In fact, the high body weight induces welfare problems, such as greatly reduced kinetic activity [47] and the increasing occurrence of podal and sternal lesions [48]. Tahamtani et al. [49] studied the walking ability of broiler chickens, showing that 77.4% of the animals exhibited an abnormal gait score and lameness. FG strains are selected for high productive performance in short timeframes (about 40 d), directing the concentration of feed resources towards muscle growth, simultaneously reducing the allocation of body resources to active behaviors, immune response and thermo-tolerance [4,50]. Moreover, the minimum slaughter age of 81 d, which is compulsory in the organic system, increases the mortality rate of this genotype, often due to sudden death syndrome [51].

For human welfare, although the C system featured a high production performance (high feed conversion, low production cost), it was penalized by its poor results in terms of income, risk category, work load and antibiotic resistance. However, in human welfare, Phi^+^ was close to OR, due to the good economic performance of both. The Phi^−^ underlined the fact that OR performed poorly in some criteria and, therefore, the Phi was higher for ONN.

In this trial, the nutritional quality of the meat was almost equivalent in the three systems compared. However, several authors underlined that organic production could improve the nutritional quality of chicken meat through the contribution of the bioactive compounds of pasture (antioxidants and polyunsaturated fatty acids) [6]. Therefore, in our study, although organic SG birds demonstrated good foraging behavior (kinetic activity and outdoor animal %), the lack of improvement in meat quality could be ascribed to the small amount of grass present in the outdoor pens.

The global index demonstrated the superior performance of both the organic systems compared with the conventional system. The comparison between the organic systems demonstrated that the use of a more suitable strain (Naked Neck;) [4] positively affects the final rank, mainly by reducing welfare problems and producing good economic and social performances.

According to the sensitivity analysis, the first position of the ONN was quite stable, in particular in comparison with the C system, which could not switch position even when changing 60% of the weights criteria, or even 76%, if we exclude an increase in the importance of the weights equal to 100%. By contrast, reversal points between OR and C were more frequent.

## 5. Conclusions

The comparison between conventional and organic systems cannot be mono-dimensional, but must be based on a global approach, in order to include all the key topics connected with the systems investigated. In this paper, we proposed a global comparison framework based on the One Welfare approach, as a theoretical framework, and on multicriteria analysis, for application in the context of commercial farms. Based on the criteria proposed, the multicriteria analysis demonstrated that the organic systems performed better overall compared to the conventional system. However, the system type alone does not provide the complete answers; rather, it needs to be considered in light of the poultry genotype. The use of the SG strain in the ONN positively affected the final rank, mainly by reducing welfare problems and producing good economic and social performances.

The stability of the results is crucial for decision support systems. The sensitivity analysis aimed to value this aspect and confirmed that the first position of the organic system using a slow-growing strain was quite stable, especially if compared to the conventional system.

Although the results are clear and stable, further studies may focus on some key aspects. For instance, positive results in terms of environmental impact could be reached modifying the feed formulation, choosing local feed and crops with high degrees of renewable energy. Along with the investigation into the feed impact, other aspects linked to the perceptions of consumers and society should be further investigated.

## Figures and Tables

**Table 1:** Mean structural and management characteristics of poultry farms.

Type	Genetic Strain	Age at Slaughtering (Days)	Indoor Area (m^2^)	Outdoor (m^2^)	Chickens Produced/Cycle (*n*)
C	Ross 308	40	3139	-	55,960
OR	Ross 308 (only females)	81	1575	38,800	9245
ONN	Naked Neck (only females)	81	840	26,800	6528

C, conventional; OR, organic Ross 308, ONN, organic Naked Neck.

**Table 2:** Main ingredients of diets (in % as average formulations for different growing periods—starter, grower, finisher).

Ingredients	Maize	Soja Defatted Meal	Whole Soja	Sorghum	Wheat	Bicalcium Phosphate	Calcium Carbonate	NaCl	Vitamin Premix
Conventional	33.0	29	-	8	25.7	1	2	0.3	1
Organic	42.5	19 *	11	-	23.2	1	2	0.3	1

* from soja panel.

**Table 3:** One welfare evaluation framework.

One welfare	**Dimensions**	**Categories**	**Criteria**
	Animal Welfare	Behavioural traits	Feeding
			Use of outdoor
			Kinetic activity
			Comfort
		Lesions	Sternal lesions
			Podal lesions
	Human Welfare	Meat quality	PUFA n-3
			PUFA n-6
		Antibiotic resistance	Antibiotic resistance
		Work safety	Risk category
			Work load
		Economic performance	Income
			Cost
		Production performance	Live weight
			Mortality rate
			Feed Conversion index
	Environment	Resp. inorg.	Respiratory inorganics
		Climate change	Climate change
		Acid. eutroph.	Acidification/eutrophication
		Land use	Land use
		Fossil fuel	Fossil Fuel

**Table 4:** Main behaviors expressed by the chickens.

Behavior Category	Behaviors	Description
Activity	Walking	Bird that moves more than three steps
	Rest	Bird that presents the body in line with the ground with an erect head and open eyes
	Roost	Bird in lying position with the ventral body region in contact with the floor
Eat	Feed	Bird that pecks inside the feeder
	Grass	Bird that presents its head down and beak in contact with the grass
	Drink	Bird that pecks the drinker
Comfort		Animal without any signs of discomfort

**Table 5:** Effect of rearing systems on the different criteria.

Criteria			C	OR	ONN
Animal Welfare					
Use of outdoors	% animals	max	-	20.2	43.7
Feeding	% budget time	min	40.8	27.7	21.7
Kinetic activity	“	max	9.6	10.9	21.8
Comfort	“	max	9.4	12.8	10.6
Sternal lesions	% animals	min	20	40	0
Podal lesions	“	min	10	27	14
Human Welfare					
Slaughter weight	g	max	2460	4650	2600
Mortality rate	%	min	3.2	5.7	4.7
Feed Conversion index		min	1.9	2.8	3.0
Income	€/per head	max	0.92	1.97	1.84
Cost	€/per head	min	1.19	1.36	1.43
PUFA n-3	% fatty acids	max	3.76	3.63	3.62
PUFA n-6	“	min	30.10	30.11	27.35
Risk category		min	5	3	3
Work load	hours per 100 head	min	1.07	5.07	5.07
Antibiotic resistance	% multi-resistant isolates	min	69.9	31.2	31.2
Environment					
Resp. inorganics		min	4.53E-04	2.37E-04	1.24E-04
Climate change		min	4.37E-05	2.39E-05	3.91E-05
Acidification, Eutrophization		min	2.29E-04	1.17E-04	4.74E-05
Land use		min	2.59E-04	3.55E-04	5.61E-04
Fossil Fuel		min	1.34E-04	1.02E-04	1.64E-04

C, conventional; OR, organic Ross 308; ONN, organic Naked Neck; PUFA n-3, Polyansatuarate Fatty Acid n-3 series; PUFA n-6 Polyansatuarate Fatty Acid n-6 series.

**Table 6:** Stability interval for organic Naked Neck (ONN) vs. organic Ross (OR), organic Ross (OR) vs. conventional (C) and organic Naked Neck (ONN) vs. conventional (C) (expressed in percentages).

Criteria	ONN/OR	OR/C	ONN/C
Animal Welfare			
Feeding	NRR	NRR	NRR
Use of outdoor	NRR	NRR	NRR
Kinetic activity	NRR	NRR	NRR
Comfort	20.71%	NRR	NRR
Sternal lesions	NRR	29.33%	NRR
Podal lesions	NRR	18.66%	38.51%
Human Welfare			
Live weight	20.71%	NRR	NRR
Mortality rate	NRR	25.48%	38.51%
Feed Conversion index	100%	100%	100%
Income	89.47%	NRR	NRR
Cost	100%	100%	100%
PUFA n-3	100%	100%	100%
PUFA n-6	NRR	29.33%	NRR
Risk category	100%	NRR	NRR
Work load	100%	22.68	30.27%
Antibiotic resistance	100%	NRR	NRR
Environment			
Respiratory inorganic	NRR	NRR	NRR
Climate change	20.71%	NRR	NRR
Acidification/eutrophication	NRR	NRR	NRR
Land use	20.71%	25.27%	29.33%
Fossil fuel	13.47%	NRR	38.51%

NRR = No rank reverse.

## Data Availability

The data presented in this study are available on request from the corresponding author. The data are not publicly available because they are sensible data of a commercial poultry company.
